# Exploring perceived factors influencing cross-system collaboration between Child welfare and behavioral Health: A qualitative study

**DOI:** 10.1016/j.chipro.2026.100336

**Published:** 2026-07-21

**Authors:** Yuanyuan Hu, Emmeline Chuang, Fawn Gadel, Jen McClellan, Alicia Bunger

**Affiliations:** a University of Minnesota, School of Social Work, Minneapolis, MN, USA; b University of California, Berkeley, School of Social Welfare, Berkeley, CA, USA; c Public Children Services Association of Ohio, Columbus, OH, USA; d Division of General Internal Medicine, Department of Internal Medicine, College of Medicine, The Ohio State University Wexner Medical Center, Columbus, OH, USA

**Keywords:** Ohio START, Parental substance use disorder, Child welfare, Behavioral health, Cross-system collaboration, Implementation science

## Abstract

**Background:**

Parental substance use disorder (SUD) significantly increases complexity and severity of child maltreatment. Sobriety Treatment and Recovery Teams (START), an evidence-based intervention, has improved unification and reduced recurrence of maltreatment risk through cross-system collaboration between child welfare and behavioral health organizations. Currently little is known about perceived factors influencing successful collaboration.

**Objective:**

This qualitative study identified factors that influenced cross-system collaboration in a state-wide implementation of an integrated social service and behavioral health intervention.

**Participants and setting:**

Embedded in an implementation study of Ohio START, this study used semi-structured small group interviews with 104 administrators and frontline providers, and structured focus groups with 26 child welfare caseworkers and family peer mentors.

**Method:**

Virtual small-group interviews were conducted between January 2020 and April 2021, and structured focus groups were conducted in February 2021. To identify barriers and facilitators of cross-system collaboration, thematic analysis was applied to transcripts, using inductive and deductive coding methods.

**Results:**

Overall, participants from both child welfare and behavioral health fields reported positive collaboration experiences and noted improvement in mutual understanding and service provision. Two main barriers emerged: misalignment of treatment requirements and expectations, and varied attitudes toward substance use and treatment. Facilitators included prior established collaboration, dedicated collaboration personnel, and supportive leadership.

**Conclusions:**

Cross-systems alignment of services and expectations, efforts to address stigma and negative perceptions, and leveraging leadership support and dedicated personnel are crucial for successful cross-system collaboration.

## 1. Introduction

Substance use disorder, particularly Opioid Use Disorder (OUD), has emerged as a significant public health issue in the United States. Substance use disorders (SUDs) among U.S. adults affect the health and well-being of millions of children living in households through direct exposure and adverse childhood experiences (Lipari & Van Horn, 2013). Parental SUD is the most prevalent risk factor for child maltreatment and has also been associated with increased complexity and severity of maltreatment (Radel et al., 2018), as well as increased risk of child protective services (CPS) involvement, medical visits, and out-of-home placements (Austin et al., 2020; Kelly et al., 2022). Although alcohol use remains the most prevalent type of substance use disorder in the U.S., significant increases over the last two decades in addiction to opiates and number of deaths from opioids has emerged as a child safety concern due to their association with increased child maltreatment and higher rates of infant foster care placements, particularly in high-poverty counties (Chapman, 2022; Freisthler et al., 2022; Mirick & Steenrod, 2016; Patrick et al., 2019). An estimated 548,000 children lived with an adult with opioid use disorder (OUD) in 2017, representing a 30% increase from 2002, and the share of children entering foster care who had parental opioid use disorder rose from approximately 2% in 2014 to 5% in 2020, underscoring a growing population exposed to parental OUD in childhood (Feder et al., 2018; Meinhofer et al., 2024). 321,566 U.S. children lost a parent (ages 18–64) to drug overdose from 2011 to 2021, with rates increasing from 27.0 to 63.1 per 100,000 children (Jones et al., 2024).

Parents with SUDs face significant barriers to treatment, including stigma, fear of custody loss, and limited availability of family-centered programs (Frazer et al., 2019; Guerrero et al., 2013; Radcliffe, 2011; Stone, 2015). They also experience challenges with perceived judgment from service providers, restrictive visitation during treatment, and a lack of childcare (Seay & McRell, 2023). Parents in rural areas encounter additional challenges, such as insufficient treatment programs, lack of facilities, and inadequate transportation compared to urban counterparts (Ali et al., 2022). Parents with SUDs involved in the child welfare system also report feeling uninformed, devalued, and disempowered, particularly when not supported by empathetic caseworkers (Colvin & Howard, 2022). For parents affected by opioid use disorder, stigma toward medication for opioid use disorder (MOUD), influenced by inadequate provider training and knowledge, and restrictive methadone regulations, serves as a further barrier to accessing needed treatment (Madden et al., 2021). At the same time, child welfare workers trying to connect parents with behavioral health treatment report significant resource shortages, limited treatment availability, long wait times for co-occurring mental health treatment, and challenges in collaborating with behavioral health providers, particularly in non-metropolitan and rural communities (Channell Doig et al., 2023; Clary et al., 2020; Orsi et al., 2021).

Given the intersection of parental substance use and child safety, cross-system collaboration between child welfare and behavioral health services can be crucial for connecting parents to treatment and improving family stability (Chaiyachati & Deutsch, 2024). A collaborative approach can reduce service fragmentation and increase access and utilization among parents with SUDs (Drabble & Poole, 2011; He, 2017; He & Phillips, 2017). Such an approach enhances child permanency and parental recovery outcomes by building shared values, fostering cross-system communication, providing comprehensive support, and ensuring priority access to treatment (Drabble, 2010; Freisthler et al., 2022). Research also suggests that inclusion of peer mentors with lived SUD recovery experience can enhance client participation in treatment and improve treatment outcomes by providing coaching, system navigation assistance, and emotional support (Huebner et al., 2018; Olding et al., 2022; Paterno et al., 2018).

### 1.1. Sobriety Treatment and Recovery Teams (START)

Sobriety Treatment and Recovery Teams (START) is an evidence-based intervention that adopts a comprehensive cross-system collaboration approach to meet the needs of families involved with child welfare and affected by caregiver substance use. START aims to increase family unification by helping families safely recover together throughout the case and/or be reunified by case closure, while reducing maltreatment recurrence for families with co-occurring child maltreatment and substance use (Huebner et al., 2021). The intervention includes multiple sequenced components (e.g., initial screening, shared decision-making, referral and contact with family peer mentors, and quick access to treatment), which are expected to be completed within specific time periods (Hall et al., 2021; Huebner et al., 2017, 2017, 2018, 2017). Alignment between child welfare and behavioral health is critical to intervention effectiveness (Wells & Chuang, 2012).

Ohio began implementing the Sobriety, Treatment, and Reducing Trauma (START) model in 2017 in direct response to the opioid crisis’s impact on children affected by parental substance use (Ohio Attorney General’s Office, 2017). As of 2025, Ohio START, a certified affiliate of the national START model, has been implemented in 56 Ohio counties. The national Sobriety Treatment and Recovery Teams (START) model has demonstrated effectiveness in improving parental access to substance use disorder (SUD) treatment, improved parental sobriety outcomes, and increased family unification for families with young children (age 0–5) (Huebner et al., 2017). Ohio START expanded eligibility to families with children of all ages (Dellor et al., 2024) and has demonstrated positive changes in parenting attitudes and practices (Yoon et al., 2024). Both the national and Ohio START initiatives include cross-system collaboration between child welfare and behavioral health providers and provide families with care planning, service coordination, wraparound support, and expedited access to behavioral health services. START creates a coordinated pathway to substance use treatment for parents through SUD screening, linkage with a family peer mentor, referral to community treatment providers for assessment and treatment, and collaborative alignment of case goals and timelines.

Successful implementation of START is contingent on effective collaboration between child welfare and behavioral health providers. However, differing treatment approaches, cultures, and priorities can make collaboration challenging. Previous studies have identified facilitators and barriers to cross-system collaboration. Challenges include funding limitations, siloed care structures, lack of training for child welfare staff, and balancing child safety with therapeutic relationships (Loch et al., 2023; Schiff et al., 2022). For example, Zilberstein and colleagues’ study (2024) found that high turnover, inadequate training, and a focus on physical child safety over psychological well-being hindered collaborative service delivery within the child welfare system.

Differences in values and beliefs about parents’ substance use behaviors also exist. For instance, behavioral health professionals are more likely to view addiction as a disease, prioritize supporting parents in treatment, feel positively about parents’ potential for treatment success, and focus on recovery than child welfare professionals (Drabble, 2007; He et al., 2014). By contrast, child welfare case managers are focused on child safety and have varying levels of SUD knowledge. They are often faced with resistance from families to self-disclose substance use, instead relying on drug screening results to detect prenatal substance exposure (Seay & McRell, 2023). These differences can influence cross-system collaboration and implementation of effective services for parents with SUD.

Although Ohio START includes multiple intervention components intended to facilitate collaboration between child welfare and substance use treatment professionals (e.g., structured screening tools, shared decision meetings, use of family peer mentors to bridge systems), it is unclear how successful these strategies were at helping overcome known barriers to cross-system collaboration. Recent implementation research on START has advanced the field by specifying cross-system collaboration strategies (e.g., co-location, expedited access agreements, shared decision-making meetings, and data sharing) that can be operationalized and potentially tested (Bunger et al., 2024); however, use of these strategies may be quite limited in practice (Chuang et al., 2024). In addition, frontline provider perspectives on their effectiveness and on factors that continue to influence collaboration even when these strategies are used.

This study extends prior work by drawing on qualitative data to explore administrator and frontline provider perspectives on factors influencing cross-system collaboration in the context of START implementation.

### 1.2. Conceptual framework

Our study is informed by the comprehensive theory of care integration. The comprehensive theory of care integration identifies three categories of factors that influence collaboration: organizational features, social features, and process activities (Chuang et al., 2025; Singer et al., 2020). Organizational features reflect the physical, legal, operational, and financial ties between organizations as well as formal policies or protocols enacted to support shared decision-making, coordination, and accountability across entities. Social features refer to shared mission, vision, culture, and quality of interpersonal relationships that can influence quality of collaboration. Finally, process activities refer to specific actions or activities taken by frontline staff to coordinate or integrate care for clients. Consistent with the comprehensive theory of care integration, this study assesses organizational features, social features, and process activities perceived by frontline providers as influencing cross-system collaboration.

## 2. Methods

### 2.1. Study design and setting

This qualitative study was part of a parent study, and program evaluation efforts examining the implementation of Ohio START (Sobriety, Treatment and Reducing Trauma) and draws on two complementary qualitative data collection approaches: (a) semi-structured small group interviews with key informants across multiple stakeholder groups and (b) structured focus groups with frontline practitioners. The parent study used a mixed-method design to identify multilevel factors influencing START implementation (Bunger et al., 2020, 2024; Chuang et al., 2024). As part of the larger program evaluation, there have also been extensive assessments of experiences, perceptions, well-being and recovery status of parents participating in the Ohio START program (Ohio, 2025; Yoon et al., 2024). All study procedures were reviewed and approved by the Ohio State University Institutional Review Board (IRB # 2017B0239). The current study complements this work by drawing on rich, qualitative data collected from child welfare and behavioral health providers to identify their perspectives on factors affecting cross-system collaboration. To strengthen transparency in sampling, data collection, and analysis, study procedures are reported in alignment with the Consolidated Criteria for Reporting Qualitative Research (COREQ) (Tong et al., 2007).

### 2.2. Data collection and sample

To triangulate perspectives on cross-system collaboration by participant role and system, we conducted semi-structured small group interviews with teams (of supervisors, leaders, and frontline professionals) drawn from a single agency or organization to understand shared collaboration experiences from the perspective of organizational units engaged in implementation. We also conducted structured focus group interviews with frontline practitioners. To reduce power differentials and support open discussion about cross-system collaboration, small group interviews were conducted separately by system (e.g., child welfare, behavioral health) and focus groups were conducted separately by professional role (e.g., caseworkers, family peer mentors).

#### 2.2.1. Small group interviews with supervisors and other leaders

Between January 2020 to April 2021, we conducted 48 small group interviews via Zoom with 104 unique supervisors and leadership staff in 17 of 30 counties in the first two cohorts of counties that volunteered to implement Ohio START. Counties included in this study constituted a convenience sample, but reflect variation in local system context (e.g., geographic diversity, child welfare agency funding, governance structure). Of the 104 unique key informants interviewed, 52 were from child welfare, 44 were behavioral health treatment providers, and 8 were regional behavioral health board representatives.

Recruitment emails were sent by the Public Children Services Association of Ohio (PCSAO) to the primary contacts at each child welfare agency, who then helped identify other eligible participants within their counties. Each participant received a $30 gift card.

Interviews averaged approximately 60 min and were facilitated by at least two research team members, all master’s- or doctoral-level investigators trained in qualitative methods and familiar with the Ohio START model. No prior relationships with participants existed beyond recruitment coordination with county contacts and professional listservs.

Interviews were conducted using a semi-structured protocol. This study focused on interview questions related to administrative aspects of collaboration, such as the relationship between behavioral health providers and child welfare providers before and after START implementation. With participant consent, interviews were audio-recorded, transcribed verbatim, and securely stored. To support reflexivity, the team also maintained brief post-interview debrief summaries (field notes), discussed assumptions related to familiarity with START and professional backgrounds (child welfare/behavioral health), and documented analytic decisions in an audit trail (Bunger et al., 2024).

#### 2.2.2. Focus groups with frontline practitioners

In February 2021, Ohio START had expanded to 36 counties and we conducted 6 structured, Zoom-based focus groups with 26 frontline practitioners working directly with START families in any county actively implementing Ohio START at the time of recruitment, e.g., caseworkers and family peer mentors.

Recruitment for focus groups occurred electronically; the research team distributed informational flyers and direct invitations through established email listservs targeting family peer mentors and START caseworkers, followed by a formal registration process for those interested in participation. Focus groups were conducted separately by roles for caseworkers and family peer mentors to explore how roles in the child welfare and behavioral health system could shape perspectives on collaboration and to foster open communication, since staff might feel reluctant to share honest views about collaboration in front of collaboration partners. These methods generated a sample of predominantly Caucasian females (Table 1) consistent with workforce demographic estimates (Armstrong et al., 2024). We presented qualitative methods, sessions, participant roles, and organization representation in Table 1.

Each focus group session lasted approximately 60 min. Two researchers familiar with the Ohio START model and trained in qualitative research methods co-facilitated each focus group. Focus group interview questions utilized in this study addressed perceived effectiveness of START, collaboration experiences between caseworkers, family peer mentors, and substance use disorder clinicians, supervision and integration processes, technical assistance received, and practical implementation challenges and recommendations. Participants provided informed verbal consent after receiving detailed information about study objectives, risks, and benefits. County-level incentives ($15 gift cards per participant) were provided, which county agencies distributed to families in need.

### 2.3. Data analysis

To manage the qualitative datasets, audio files and transcripts were stored securely, and transcripts were de-identified prior to analysis. Transcripts and debrief summaries were imported into NVivo to support data organization and retrieval. Thematic analysis was used to analyze small-group interview transcripts, following Braun and Clarke’s six-phase framework (Braun & Clarke, 2006). This process involved data familiarization, generating initial codes, searching for themes, reviewing themes, defining and naming themes, and producing the report. Both inductive and deductive coding approaches were used to allow new insights to emerge while maintaining alignment with the research focus. Building upon the existing codebook developed in the parent study, this analysis specifically emphasized collaboration between child welfare and substance use treatment systems and generated additional codes to reflect new themes emerging from the frontline practitioner focus groups. The primary coder YH conducted the initial coding using NVIVO 14, with a second coder EC reviewing the results and providing feedback. The primary coder first read and re-read the interview transcripts thoroughly, making preliminary notes and early impressions. Next, the coder focused on identifying factors influencing cross-system collaborations, leading to the emergence of initial codes. The initial codes were discussed and refined with the second coder, resulting in the development of a codebook through iterative cycles. Themes were identified to capture patterns of barriers and facilitators of cross-system collaboration within the implementation of Ohio START by examining the codes. Codes were organized into broader categories addressing the research question. For example, the code “joint supervision from child welfare and behavioral health” aligned with organizational features, whereas the codes “attitude towards medication treatment for SUD; stakeholder buy-in of START model” reflected social features. Codes related to providers’ different perceptions and attitudes, which reflected social features, were combined into the theme “varied knowledge attitudes toward substance use and treatment.” Themes were reviewed to ensure they reflected the data accurately and to understand the relationship between themes and the implementation of cross-system collaborations. After reaching an agreement, the primary coder applied the codebook to the rest of the interview transcripts. Analytic outputs (e.g., theme summaries and quotes) were organized by stakeholder systems and roles (child welfare, behavioral health treatment, behavioral health boards, FPMs) to compare perceived barriers and facilitators. To enhance credibility, we triangulated coders’ perspectives and maintained an audit trail of coding decisions and codebook iterations. Interview and focus group transcripts were not returned to participants for comment/correction due to workload and confidentiality constraints. However, to strengthen pragmatic validity of interpretations, aggregated thematic results were shared with PCSAO collaborators for feedback and validation. Finally, to personalize quotations, we have assigned first-name pseudonyms to all participants in the Results section.

## 3. Results

Overall, in the early implementation phase, participants from both child welfare and behavioral health fields reported generally positive collaboration experiences and noted improvement in mutual understanding and in service provision for parents with SUDs since the implementation of Ohio START. In general, START was praised for establishing a framework that facilitated cross-system collaboration and alignment of mutual goals. Participants also identified multiple, specific barriers and facilitators to cross-system collaboration in the context of START implementation, including organizational features (e.g., pre-existing collaborative infrastructure and dedicated personnel facilitating collaboration) and social features (e.g., alignment issues in service requirements and expectations, cross-sector variability in knowledge and attitudes, and supportive and engaged leadership) that shaped cross-system collaboration between child welfare and SUD treatment partners (see Table 2).

### 3.1. Barriers

Participants described two main barriers to cross-system collaboration and effective implementation of START: perceived misalignment of service requirements and expectations between child welfare and behavioral health systems and varied cross-system knowledge and attitudes among stakeholders.

#### 3.1.1. Misalignment of service requirements and expectations

In the early phases of START implementation, interview participants reported that behavioral health providers and child welfare services had different opinions about the types of SUD treatment approaches that were appropriate, expectations regarding treatment timelines, and information that should be shared for high-quality collaboration. This pattern reflects the social features of integration, related to cross-system differences in shared norms, values and behaviors. Perceived misalignment of service requirements and expectations between child welfare and behavioral health providers were attributed to system differences in treatment models, service timelines, and information needs, and were highlighted as requiring close attention to organizational policies and protocols subsequently enacted to support cross-system collaboration. For example, multiple participants from the child welfare agencies noted that the types of treatment approaches used by behavioral health providers could influence their perceptions about the quality of collaboration and whether parents’ service goals were met. Some behavioral health providers offered services rooted in the 12-step program and abstinence-based approaches, while others adopted harm reduction approaches. Child welfare staff stressed the importance of collaborating with behavioral health providers who delivered treatment approaches and models that aligned with state and federal requirements for child welfare cases, and noted that the use of harm reduction approaches was more likely to lead to friction with child welfare. During the early phases of Ohio START implementation, some child welfare caseworkers described harm reduction approaches as incompatible with child welfare system permanency timelines and the need to demonstrate client progress toward abstinence for program graduation. One child welfare staff member, Mary, attributed this friction to fundamental differences in child welfare services and recovery timelines:

“I think the biggest challenge is that children’s services timelines and recovery timelines are dramatically different. And sometimes behavioral health providers don’t understand that ... ... But in children’s services, we also have very strict guidelines and very strict rules. So, harm reduction is, I’m a huge personal fan of harm reduction policies, but harm reduction doesn’t work in children’s services. You have to be completely clean and sober to get your children back.”

Securing timely substance use treatment appointments emerged as another challenge in aligning services, leading to delays in the expedited service timeline required by the START model. The existing timeframe for clients accessing SUD service and limited provider capacity in some communities were perceived as creating barriers to addressing the immediate needs of clients referred by child welfare. As one child welfare caseworker, Diane, noted, “*It’s like eight weeks to get in for their first appointment and we can’t do that. They [parents] can get an intake with [behavioral health provider] and see a behavioral or a community behavioral health worker, but they can’t see their medication-assisted treatment (MAT) doctor, which is supposed to be within like four days I think for START.* ”.

Child welfare case workers also reported challenges obtaining timely and comprehensive information on parents’ SUD treatment attendance, compliance, and progress. Child welfare staff expected clients to engage in regular, supervised drug screenings and participate in services to show progress toward abstinence as part of their role to ensure child safety. Several caseworkers attributed difficulties obtaining timely supervised drug screening results from behavioral health providers to the two systems having different treatment priorities and confidentiality considerations. Several child welfare caseworkers reported greater difficulty obtaining timely and comprehensive information on parents’ SUD treatment attendance and compliance, including drug screening data, from behavioral health providers that endorsed a harm reduction approach. Child welfare workers expressed concerns about what they perceived as a lack of accountability from these providers, citing potential risks to clients and families. Common complaints about providers using the harm reduction model included not conducting drug tests, lack of supervision during drug tests, and insufficient monitoring of medication-assisted treatment (MAT) medication. One caseworker, Alex, reported:

“ ... the main hospital provider out here does not supervise drug screens. They kind of just give people second chances a lot. When we tell them [parents are] screening positive, they don’t believe us. So we pretty much just work with one [other provider]. ”

A child welfare caseworker, Sam, discussed the influence of the harm reduction model on drug screening and how she managed this issue:

“I’ll always ask for urinary drug screens ... I always ask for any concerns that they had, update on recommendations ... Because although it’s like how the clinician is looking at a parent’s say drug use and their addiction, they may be looking more at a harm reduction where we may not necessarily be able to do that, but I can take their professional opinion and kind of use it with my families.”

#### 3.1.2. Cross-system variability in knowledge and attitudes

The majority of participants from both child welfare and behavioral health systems described motivation to work together with the shared goal of child safety, expedited access to SUD treatment, and family preservation. Participants perceived Ohio START as helping to facilitate meaningful communication between stakeholders and as enabling them to address cross-sector collaboration challenges and bridge gaps in care. These findings aligned with the social features of integration and reflected greater alignment in shared goals, priorities, and underlying values across systems, as well as strengthened cross-system working relationships. Child welfare staff reported that some behavioral health providers demonstrated a strong understanding of child welfare principles, particularly the importance of assessing risk and prioritizing children’s safety. A behavioral health provider, Morgan, discussed their experiences collaborating with child welfare staff: *“There has been a definite improvement [following START]. The frequent communication helps child welfare understand better where that client is actual in their recovery, ... what their clients’ mental health needs are and how those impact their ability to parent safely.”*

However, in the early phase of establishing cross-system collaboration, participants noted that varied understandings and attitudes towards each other’s service systems still existed, which in turn could affect the development of trust, care coordination and information exchange between frontline providers. Some child welfare caseworkers held negative views towards substance use and any associated involvement with the criminal justice system, and towards family peer mentors with substance use histories. When discussing clients in the START program, a minority of caseworkers used discriminatory and derogatory language, attributing substance use behaviors to personal choices, lack of willpower, or moral flaws.

In contrast, other child welfare caseworkers demonstrated deeper understanding and endorsement of the disease model for substance abuse. One caseworker, Jesse, called for more community education to change attitudes toward addiction, shifting from viewing SUD as a choice and parents choosing addiction over their children to accepting it as a disease: *“And as far as the community goes, it’s a matter of education, education, education* .... *I mean people’s attitudes towards addiction. They may consider substance use that it’s a disease, but then the attitude can flip back to thinking it’s a choice and they’re choosing addiction over their children. That’s an ongoing battle.”* START caseworkers and behavioral health providers reported that some staff in other child welfare agencies not involved in START held negative views toward substance use, leading to resistance to Ohio START implementation. Initially, some behavioral health providers expressed concerns about bias, stigma, and a lack of understanding of substance use within child welfare organizations, affecting both family peer mentors and clients. After START implementation, however, most staff came to appreciate family peer mentors (FPMs)’ knowledge and lived experiences over time, finding them valuable even beyond the START program. One caseworker, Robin, disclosed concerns among some of her agency staff about bringing in FPMs but shifted their attitude after witnessing positive impacts:

“Most of my coworkers were really accepted to it, but there’s still some of them that are still small-minded and what they think about addiction is very negative and it drives me insane, but we’re approaching that one step at a time. But I think they were very accepted to [FPM] now. They really value her input. I see them going and asking her questions when they have other clients. And it’s pretty cool to watch it grow.”

Varied attitudes towards treatment modalities also affected the extent to which child welfare frontline workers respected clients’ autonomy in making treatment decisions. While the majority of child welfare staff respected clients’ choice of behavioral health provider, a few caseworkers reported that they viewed clients preferring MAT services as taking the “easy way out” and not putting in effort (even though the START model supports the use of MAT when clinically indicated). These caseworkers indicated that they would not recommend or would oppose MAT, believing it did not hold clients to high expectations. One caseworker, Dana, discussed her opinion regarding why clients chose MAT:

“I mean, I think there’s some clients that they don’t think they need the level of treatment that they probably actually do need. And there are some providers that are minimalists and they give them their MATs and not a lot of treatment. So of course to the client that doesn’t really want to put a whole lot of effort in that seems fantastic that they can cross off their boxes and work with us.”

Behavioral health providers’ knowledge and perceptions about child welfare practice also influenced how they collaborated with child welfare workers. Difficulties in timely information exchange was partially attributed to behavioral health providers’ mistrust and misperceptions about child welfare systems, leading to resistance to reporting clients’ regular drug screening and sharing comprehensive behavioral health treatment data with caseworkers. Some behavioral health providers reported being influenced by their clients’ accounts of negative experiences with the child welfare system and were concerned about the potential negative impacts of releasing clients’ information. They had to balance confidentiality with sharing requested information, such as drug screening results and treatment data. In response to mistrust, child welfare workers emphasized that START was shifting the traditional approach and working hard to keep children in their homes. A child welfare leader, Casey, having learned from prior experiences hiring someone strongly opposed to MAT, updated their hiring requirements to ensure FPM candidates are open to multiple paths to recovery:

“So one of shouldn’t say one of our biggest issue and concern with our first peer mentor was her beliefs on MAT, which she was strongly opposed to, and very vocal about her opposition to that, and her feelings that parents who are on MAT should not parent their children, and that they should be incarcerated and have their children permanently taken away ... So she made her feelings very clear to the families that she was working with, and very clear to the treatment providers. And I think that was a big piece for us when we hired the second one.”

One supervisor, Susan, disclosed initial mistrust due to hearing client-reported experiences, which led to stereotypes towards child welfare systems prior to the collaboration. However, they developed a deeper understanding of child welfare practices and perspectives with Ohio START implementation:

“It’s one-sided because we’re hearing it from clients. We hear it so often from so many clients that children services is not always on their side. That while, yes, it is to protect their children, that a lot of times they’re not given any sort of chances ... Yeah, rather than like judging that they’re [child welfare] not doing their job, and they’re not being fair. Maybe just understanding them as a whole and as an agency, that they’re struggling with things too. Maybe if you put a face to that children services worker name, or the therapist, and maybe you’re less apt to judge them, or have negative feelings about them.”

### 3.2. Facilitators

Participants identified three main facilitators of cross-system collaboration between frontline child welfare and behavioral health professionals (i.e., interpersonal integration): prior collaborative experiences; use of designated personnel to help establish and maintain collaborative relationships and facilitate ongoing activities; and supportive leadership, reflecting organizational features.

#### 3.2.1. Pre-existing collaborative infrastructure

Child welfare agencies in most counties had experience partnering with behavioral health providers before START implementation, reflecting an organizational feature of integration by providing an existing cross-system foundation on which partnerships could be built. These relationships were described as crucial for identifying potential behavioral health partners, establishing formal and informal agreements related to START, and ensuring a seamless transition into START. A child welfare director, Ellen, highly praised her agency’s strong, prior relationship with regional behavioral health providers, which was perceived as enabling prompt buy-in to START and prompt implementation of the model in her county:

“We have always had a very strong relationship with behavioral health [organization name]. So when we raised our hands so to speak and said we would like to be part of Cohort 1, we’d immediately thought of [organization name] as we were writing a [request for proposal], as we were thinking about how do we partner for the peers with behavioral health.”

Behavioral health providers emphasized the importance of ongoing collaborative relationships to increase their familiarity with the service scope and working culture of child welfare agencies. These partnerships have evolved over time, leading to strong motivation to continue collaboration, effective coordination, and readiness for change. Involvement in START has further strengthened these relationships and enhanced services provided to the community. A behavioral health professional, Lee, shared:

“So I guess it’s important to bring to light the fact that my position with the agency before with [behavioral health provider] before we started the partnership with Ohio START with our child welfare agency, I was one of the folks in the county that works specifically with child welfare cases ... .... So [behavioral health provider] and Children’s Services in our area were pretty close anyway, and I feel like we spent a good number of years cultivating a connection and dynamic between the two agencies that just allowed us to work very closely. I know that Ohio START had a pilot program several years ago ... ...so [behavioral health provider] know that that framework was there.”

Staff from a few child welfare agencies that attempted to engage behavioral health providers without prior relationships reported difficulties in securing their commitment due to the requirements for weekly information exchange. These challenges, along with policy changes in the state, delayed the implementation process, hindered the timely provision of services for families, and ultimately affected implementation fidelity.

“They [behavioral health provider] didn’t really understand what the expectations were or they just had enough for that person to do on their own or just not a good working relationship ... And when that change in Medicaid happened, it caused one of our major service providers that we used for adults and kids with regards to mental health they ended up closing ....So I think it’s just going to take some time but we’ll get there.”

#### 3.2.2. Dedicated personnel facilitating collaboration

Some child welfare agencies and behavioral health providers had dedicated coordinators to facilitate collaboration for Ohio START. These coordinators played a pivotal role in ensuring seamless information exchange and communication between child welfare and behavioral health providers. They managed the referral process and coordinated joint meetings, ensuring all professionals and families were aligned. Their efforts bridged the gap between agencies, guaranteed that families received timely and comprehensive support, and enhanced the efficiency and effectiveness of collaborative efforts. Dedicated personnel represented organizational features of integration by assuming formal cross-agency roles and establishing standardized information-exchange and referral channels, thereby strengthening structural and functional linkages between child welfare and behavioral health providers. An administrator, Taylor, discussed the benefits of having dedicated personnel within child welfare agency to streamline the referral process and connect clients to child welfare caseworkers:

“The supervisor has the referral specialists in her unit as well, because they can identify this as a possible case, and will let the worker know and let Ohio START Case Worker 1 or Ohio START Case Worker 2 know.”

One county contacted an external agency as the primary point of contact for all behavioral health assessment and treatment referrals, utilizing the external agency’s network of business agreements:

“We’ll refer everyone to START whether they’re engaged in services are not. Just so that we have a point of contact at [organization name]. So they can help if we have problems getting reports, because sometimes they’re not very timely with reports, some of our service providers, they can also help facilitate and troubleshoot issues or problems that come up with treatment.”

With knowledge of both systems and the START model, dedicated personnel overseeing collaboration facilitated timely reporting between the two parties involved in collaborations. These efforts ensured the alignment of expectations and streamlined information exchange, contributing to the successful implementation of Ohio START. A few child welfare supervisors mentioned that having designated personnel in charge of collaboration was their next step or future priority:

“I would have a designated counselor ... say, I have someone who’s in need of treatment, whether that be an assessment, or detox, or inpatient treatment, and they could get them in quickly for an assessment and they would understand. They would already know what the Ohio START model is and I wouldn’t have to spend so much time explaining what this model is and what we’re trying to do.”

#### 3.2.3. Supportive and engaged leadership

Supportive leadership in both systems, at both the senior leadership and supervisor level, was perceived as critical for establishing and maintaining strong relationships between child welfare agencies and behavioral health providers. This facilitator reflected an organizational feature of integration, as leadership shaped its structural and functional aspects. Participants noted that leaders and supervisors helped build collaboration and trust across the two systems by proactively reaching out to partners, fostering connections, and ensuring clear expectations through contracts or agreements. Active communication between leaders and supervisors across systems facilitated information exchange, particularly regular updates and progress monitoring of families. When asked about unique contributions of senior leaders as opposed to supervisors, participants described top-down support from senior leadership as essential for initiating conversations about START and committing to agreements. Supervisors contributed to day-to-day problem-solving by promptly addressing implementation challenges. A leader, Ava, from the regional behavioral health board spoke highly of the relationship between directors at the behavioral health and child welfare agencies:

“I think that the biggest positive that we have had is that our clinical director, and their director, are friendly, and they get along. That has been a huge positive, because anytime there’s been any sort of animosity towards clinicians, or anything, they have felt like they can go to each other and say, “Hey, your person’s doing this. Like what’s up,” and it gets addressed. Neither one of them I think has felt negative towards it.”

Supervisors were described as playing key roles in leading implementation and establishing clear expectations within their agencies to deliver the program. Regular supervision and support between supervisors and their team members provided instruction and guidance for frontline workers, fostering teamwork despite varying organizational contexts and cultures. One behavioral health supervisor, Jasmine, shared the responsibilities of her role:

“Supervisors spend a lot of time teaching them how to work on goals and make goals and meet that requirement. I do hold them to a pretty high standard in terms of their care notes. They don’t have the four year degrees or the graduate degree that’s necessary to write a clinical note but I do provide them a lot of the tools. We follow those, an [documentation rules]. Every interaction they have with the family, they have to identify which of [documentation rules] they’re fulfilling and then they have to speak to that in their care notes.”

Family peer mentors in several counties reported that receiving joint supervision from more than one organization was beneficial for effective implementation and enhancing their professional knowledge and skills. FPMs had the opportunity to present cases and receive clinical feedback or approval from different supervisors: “I was getting supervision from child welfare and behavioral health. Supervision consists of a clinical meeting on Thursdays for a few hours that we do every week. And that’s cool because I’m able to bring cases. Every clinician has to bring two to three cases and just present them ... and then we might get either some feedback or suggestions from all the clinical staff at the table or get approvals for stuff.”

## 4. Discussion

This study explored the experiences and perceptions of child welfare workers and behavioral health providers at both administrative and frontline levels in the early stages of implementing Ohio START, an intervention focused on improving alignment between child welfare and behavioral health providers. Informed by the comprehensive theory of care integration, our findings highlighted how organizational and social features as well as process activities shape collaboration between child welfare and behavioral health providers to enhance care for families involved with child welfare and affected by parental SUD. Our results highlight that during early implementation, persistent barriers remain, especially those related to the social features of collaboration including different expectations, misaligned system missions, and variability in knowledge and attitudes. Barriers in the social features of integration are especially salient during early implementation, including misaligned service requirements and expectations and variability in knowledge and attitudes. These normative misfits are rooted in structural and functional differences between the two systems and may impede alignment around service expectations and shared interpretations of progress. However, high-quality organizational features that link outer and inner context, including prior partnerships, dedicated referral personnel, and supportive leadership might bolster collaboration, thereby reinforcing interpersonal and process integration in cross-system collaboration. It might be that these organizational features provide a foundation for frontline professionals as they work through their differences, and build stronger collaboration over time.

With regards to cross-system collaboration, study findings highlight the tensions that can arise during early stages of efforts to align systems that may have very different values, goals, and service timelines that shape the social features of collaboration. In particular, challenges can arise if professionals operating under very different service paradigms are asked to frequently interact and collaborate, and they have only a limited or erroneous understanding of one another’s systems. Our results showed that, during early implementation, social aspects of integration often became challenging. Child welfare and behavioral health partners often approached work with different institutional requirements, service timelines, and understanding of progress. During early implementation phases, organizations needed to build alignment across the two systems by shaping structural and functional integration (e.g., governance arrangements, referral pathways, information-sharing protocols) in ways that fit outer-context constraints (e.g., statutory mandates, confidentiality regulations, service capacity) and inner-context conditions (e.g., organizational routines, workforce norms). When these organizational features were underdeveloped, social aspects of integration were more likely to be interrupted, impeding cross-system coordination, especially when collaboration was frequent and under time pressure. For example, prior research has described the child welfare system as endorsing a “linear*”* substance use treatment model while behavioral health systems more often recognize relapses as a part of recovery (Adlin Bosk et al., 2017). When these tensions are not resolved, this can result in unrealistic recovery expectations and a lack of person-centered care (Colvin & Howard, 2022). Participants in our study noted that Ohio START’s family-centered approach has the potential to bridge the divide between systems by focusing professionals from both systems on the shared goals of child safety, parental recovery, and family reunification. However, our study found that at the time of data collection, some agencies still struggled to align child welfare and SUD treatment quickly enough to ensure fidelity of implementation and adherence to child welfare policies. Existing literature reports a tension between child welfare and harm reduction services due to their different focuses and safety regulations (Hawk et al., 2017; Shlonsky & Wagner, 2005). Substance use clinicians endorsing a harm reduction approach often showed hesitancy in raising parenting concerns to preserve therapeutic alliances, due to confidentiality constraints and misaligned timelines with child welfare (Padilla-Petry et al., 2025). With time and sustained collaboration, it is possible that child welfare and behavioral health professionals will resolve different views on treatment modalities and requirements, improve coordination, and expedite parents’ access to treatment. Providing cross-training and supervision to clarify roles and establishing information-sharing and joint decision-making tools could potentially reduce tension between child welfare and harm reduction services so decisions remain child-centered (Hawk et al., 2017; Padilla-Petry et al., 2025; van Boekel et al., 2013).

The findings also revealed that frontline social service and behavioral health providers’ knowledge of collaborating systems and attitudes towards SUD and treatment also influence the social features of collaboration. Although some participants reported that their knowledge and understanding of their partner organizations deepened over time, enabling them to improve collaboration, other participants still struggled. In particular, behavioral health providers reported feeling as though stigma toward SUDs within the child welfare system limited their ability to collaborate and implement Ohio START. These stigma dynamics can be conceptualized as social features of integration, particularly challenges in normative integration involving shared beliefs and cultural expectations about SUD and recovery that undermine interpersonal integration (e.g., communication and teamwork). These social barriers can be reinforced by structural stigma embedded in organizational policies, regulatory requirements, and legislation. Collectively, stigma operating across these levels can impede information sharing and delay coordinated care processes. Other studies highlight how stigma can result in viewing substance use as a moral failing rather than a medical condition requiring treatment (Zwick et al., 2020). This stigma leads to preferences for coercive treatment and restricted access to recovery support (Paquette et al., 2018; Stopka et al., 2024; Yang et al., 2017). Health professionals with negative attitudes toward clients’ substance use behaviors often demonstrate reduced empathy, impacting treatment outcomes (van Boekel et al., 2013). Despite the efficacy of MAT for opioid use disorder, skepticism remains due to misconceptions about its efficacy and concerns when clients do not receive clinical services along with MAT (S. Cooper & Nielsen, 2017; Hall et al., 2016; Wakeman & Barnett, 2018; Witte et al., 2021; Zwick et al., 2020). Our study highlighted that stigmatizing attitudes toward SUDs and MAT among caseworkers could potentially impede collaboration, reducing the fidelity of implementation.

In our study, behavioral health providers also had differing knowledge, expectations, and understanding of child welfare practice which affected their collaboration. Similar to other studies, behavioral health providers often struggled to comprehend the complexity and urgency of the child welfare system, leading to role confusion and fragmented efforts. This limited understanding contributes to communication barriers, mistrust, and sometimes withholding critical information, impeding timely case progression in alignment with child welfare goals (Hwang et al., 2017). Additionally, discrepancies in organizational structures and philosophical approaches hinder their ability to synchronize efforts with child welfare workers, thereby affecting the effective identification and mitigation of safety risks (Boel-Studt et al., 2022; Colvin & Thompson, 2020). Ohio START training includes modules on child welfare practice for behavioral health professionals, and SUD treatment for child welfare case workers (that also introduces the efficacy of MAT) to bridge gaps in understanding among professionals. These trainings for professionals are intended to correct misperceptions about SUD treatment and child welfare practice and reduce stigma about parental substance use disorder. The ongoing challenges that our participants highlighted suggest the importance of continuous training, strong organizational leadership and supervision that reinforce the values of recovery, and sustained collaboration to help frontline professionals develop a common understanding of each system’s goals and frameworks (R. E. Cooper et al., 2024).

Consistent with the comprehensive theory of care integration, organizational features emerged as key facilitators for strengthening collaboration. The parent study for Ohio START implementation identified multi-level implementation strategies shifting organizational features of integration to facilitate collaborations between child welfare and behavioral health systems, including co-locating staff, contracting with providers for expedited service access, clear referral protocols, shared decision-making and data sharing (Bunger et al., 2024). In other studies of cross-system interventions in child welfare, strong leadership support, consistent training, clear guidance, tools and resources, regular communication, and policy support are also important facilitators (Gopalan et al., 2021; Winters et al., 2020; Zilberstein et al., 2024). Our study expands on this knowledge by emphasizing the importance of prior collaboration experiences, designated staff, and supportive leadership, which reflect organizational features. Previous cross-system collaboration experiences among our participants helped them understand each system’s culture, requirements, and client needs, preparing them to align services and communication effectively. Creating designated positions or contracting with external agencies managing collaboration could also help follow clients through the START care pathway, especially considering the high workload on both sides. Leadership and supervisors with strong commitment played a key role in identifying partners, participating in implementation stages, and ensuring successful execution within their agencies. Notably, our results did not surface factors related to process activities (e.g., cross-system shared decision-making meetings). One plausible explanation could be timing, since the parent study collected data in the early stages of implementation. Participants may have been primarily focused on establishing the organizational and social foundations of collaboration (e.g., structures, trust, and shared expectations) before process activities that facilitate effective cross-system coordination became routine practice.

### 4.1. Future directions

At the time of this study, child welfare and behavioral health professionals were in the process of implementing Ohio START with families, working through collaboration challenges, and improving fidelity. This progress was supported by strong statewide implementation support including comprehensive training, technical assistance, opportunities for shared learning, a program-specific data system to inform decision making, and tailored tools like a multi-stage hiring guide for family peer mentors. Indeed, the statewide infrastructure provided is consistent with many best practices and principles for implementation support (Albers et al., 2021). Despite the robust implementation support provided, professionals encountered collaboration challenges. This difficulty might not necessarily reflect inadequacies in the support system but rather underscores the inherent complexity of the work. It is essential to recognize that time and persistence are necessary for individuals to navigate and overcome these challenges effectively. Future studies are needed to understand the speed and pacing of collaboration and implementation to set reasonable expectations for progress, especially when partners do not have prior experience working together.

Developing and testing strategies to improve collaboration during implementation of cross-system interventions could also be useful for helping agencies work through specific partnership difficulties. For instance, results from the parent study suggested that formal collaboration support (e.g. formal contracts, referral protocols, joint supervision arrangements, shared decision-making meetings, and engaging third party facilitators) were rarely used among implementing counties, because local leaders were not aware of these strategies. These findings point to an actionable implementation target: increasing leaders’ knowledge of collaboration-support strategies and building local capacity to select, tailor, and routinize them in response to specific barriers (e.g., role ambiguity, referral bottlenecks, competing mandates). This analysis also underscores the necessity for comprehensive policies supporting information-sharing guidelines that protect client confidentiality. Future studies are needed to examine the impact of these strategies on the quality of collaboration, implementation fidelity, and outcomes for parents and children and test whether effects vary by baseline partnership strength and outer-context constraints (e.g., workforce shortages, treatment availability).

### 4.2. Limitations

This study has several limitations that should be acknowledged. First, the interview data were collected as part of the parent study examining the implementation in a single state during early implementation stage. The unique context of Ohio’s county-administered structure and strong state-level support may impact the generalizability of results to other implementations of cross-system collaborations, considering variations in regulation, funding environment, and workforce in other states. This study aimed to understand varying perceptions of cross-system collaborations within specific counties and the findings should not be used to draw inferences regarding all child welfare workers and behavioral health providers. Future comparative research across states should test the generalizability of these findings and examine whether implementation strategies targeting similar barriers improve cross-system collaboration. Second, frontline staff may have experienced discomfort or reluctance to disclose their views candidly when being interviewed in the same small group with administrators, which may have increased social desirability bias, particularly when stigmatizing attitude, or adherence to intervention. Notably, despite being common system barriers to collaboration in human services (Sandfort, 1999), issues related to power dynamics, unequal access to resources, and policy constraints did not emerge in conversation. This risk may have been heightened in small-group formats that included mixed organizational roles, potentially discouraging candid discussion of sensitive topics such as power differentials, resource inequities, or policy constraints. We attempted to mitigate this limitation by allowing frontline staff to discuss their experiences openly and freely (where case workers and family peer mentors were separated into distinct groups). To further minimize biases, we used a trained moderator, emphasized confidentiality, and triangulated findings in role-specific focus groups and mixed-role sessions. Nonetheless, future studies might probe on these sensitive topics. Third, the study focused solely on the perceptions of providers, offering a limited understanding of the client’s views. Given families’ central roles in care navigation, engagement, and decision-making, the absence of caregiver perspectives may have limited our ability to identify determinants related to family burden, perceived coercion, and family-experienced stigma, factors that can critically shape whether cross-system coordination is experienced as supportive and coherent. Evaluation of parent graduates of Ohio START conducted by Ohio University reported that compassionate, highly accessible family peer mentor supports and improvements in parenting and well-being, providing valuable insights into parent experiences (Ohio, 2025; Ohio, 2023). Future studies should employ mixed-method approaches to integrate both qualitative and quantitative data, effectively connecting identified facilitators and barriers to implementation and client-level outcomes, thereby providing a more robust and holistic understanding of the initiative’s impact. Fourth, data were collected during early implementation, when workflows, referral pathways, and interagency relationships were still developing and adjusting (including during the COVID-19 period). Some barriers may therefore reflect transitional implementation conditions rather than stable features of mature cross-system collaboration, and certain facilitators (e.g., routinized coordination practices, sustained leadership alignment, and long-term trust-building) may be underrepresented.

## 5. Conclusion

The findings provided a nuanced understanding of factors influencing collaborations between child welfare and behavioral health treatment systems. Results highlighted the importance of improving the alignment of social features of collaboration such as expectations between the two systems, addressing stigma and negative perceptions towards substance use behaviors and MAT, and developing strategies to enhance the quality of collaboration. Organizational features, including strong leadership, experiences of cross-system collaboration and designated personnel managing the collaboration could facilitate better implementation. This study offered valuable insights for implementing cross-system collaboration between social services and behavioral health service systems and could inform future studies to build effective implementation strategies. The study also provides actionable guidance to inform state and county protocols, contracting, performance monitoring, and evaluation of integration. In sum, the results offer a clear path for immediate practice change and for policy and research to scale and sustain cross-system collaboration.

## Figures and Tables

**Table 1:** Sociodemographic demographics of participants.

Small Group Interviews (n = 104)		Focus Groups (n = 26)	
	n		n

*System*		*Role*	
Child Welfare	52	Child Welfare Caseworkers	17
Behavioral Health	44	Family Peer Mentors	9
Behavioral Health Boards	8		
*Sex*		*Sex*	
Female	90	Female	25
Male	4	Male	1
Not reported/unknown	10	Not reported/unknown	0
*Race/Ethnicity*		*Race/Ethnicity*	
Caucasian	57	Caucasian	19
Black/African American	5	Black/African American	1
Other	3	Other	0
Not reported	39	Not reported/unknown	6

**Table 2:** Barriers and facilitators with representative quotes.

Barrier (−)/Facilitator (+)	Type of Integration	Representative Quote

Alignment issues of service requirements and expectations (−)	Social feature	*“I think the biggest challenge is that children’s services timelines and recovery timelines are dramatically different. And sometimes behavioral health providers don’t understand that ... ... But in children’s services, we also have very strict guidelines and very strict rules.*
Cross-sector variability in knowledge and attitudes (−)	Social feature	*Most of my coworkers were really accepted to it, but there’s still some of them that are still small-minded and what they think about addiction is very negative and it drives me insane, but we’re approaching that one step at a time. But I think they were very accepted to [FPM] now.*
Pre-existing collaborative infrastructure (+)	Organizational feature	*We have always had a very strong relationship with behavioral health [organization name]. So when we raised our hands so to speak and said we would like to be part of Cohort 1, we’d immediately thought of [organization name] as we were writing a [request for proposal], as we were thinking about how do we partner for the peers with behavioral health.*
Dedicated personnel facilitating collaboration (+)	Organizational feature	*The supervisor has the referral specialists in her unit as well, because they can identify this as a possible case, and will let the worker know and let Ohio START Case Worker 1 or Ohio START Case Worker 2 know."*
Supportive and engaged leadership (+)	Organizational feature	*Supervisors spend a lot of time teaching them how to work on goals and make goals and meet that requirement. I do hold them to a pretty high standard in terms of their care notes.*

## References

[R1] Adlin Bosk E, Van Alst D, & Van Scoyoc A (2017). A chronic problem: Competing paradigms for substance abuse in child welfare Policy and practice and the need for new approaches. British Journal of Social Work, 47(6), 1669–1685. 10.1093/bjsw/bcx095

[R2] Albers B, Metz A, Burke K, Bührmann L, Bartley L, Driessen P, & Varsi C (2021). Implementation support skills: Findings from a systematic integrative review. Research on Social Work Practice, 31(2), 147–170. 10.1177/1049731520967419

[R3] Ali MM, Nye E, & West K (2022). Substance use disorder treatment, perceived need for treatment, and barriers to treatment among parenting women with substance use disorder in US rural counties. The Journal of Rural Health, 38(1), 70–76. 10.1111/jrh.12488. Official Journal of the American Rural Health Association and the National Rural Health Care Association. 32613709

[R4] Armstrong JM, McCarthy M, Ringeisen M, & Dolan M (2024). Snapshot of the child welfare workforce from 2021 to 2022: NSCAW III workforce Study design, data collection, and sample characteristics. OPRE report #2024–025. Washington, DC: Office of planning, research, and evaluation, administration for children and families. U.S. Department of Health and Human Services.

[R5] Austin AE, Lesak AM, & Shanahan ME (2020). Risk and protective factors for child maltreatment: A review. Current Epidemiology Reports, 7(4), 334–342. 10.1007/s40471-020-00252-3 PMC8205446 34141519

[R6] Boel-Studt SM, Flynn HA, Deichen Hansen ME, & Panisch LS (2022). Enhancing behavioral health services for child welfare-involved parents: A qualitative study. Journal of Public Child Welfare, 16(5), 607–631. 10.1080/15548732.2021.1942389

[R7] Braun V, & Clarke V (2006). Using thematic analysis in psychology. Qualitative Research in Psychology, 3(2), 77–101. 10.1191/1478088706qp063oa

[R8] Bunger AC, Chuang E, Girth A, Lancaster KE, Gadel F, Himmeger M, Saldana L, Powell BJ, & Aarons GA (2020). Establishing cross-systems collaborations for implementation: protocol for a longitudinal mixed methods study. Implementation Science: IS, 15(1), 55. 10.1186/s13012-020-01016-9 PMC7364639 32677987

[R9] Bunger AC, Chuang E, Girth AM, Lancaster KE, Smith R, Phillips RJ, Martin J, Gadel F, Willauer T, Himmeger MJ, Millisor J, McClellan J, Powell BJ, Saldana L, & Aarons GA (2024). Specifying cross-system collaboration strategies for implementation: A multi-site qualitative study with child welfare and behavioral health organizations. Implementation Science, 19(1), 13. 10.1186/s13012-024-01335-1 PMC10863233 38347639

[R10] Chaiyachati BH, & Deutsch SA (2024). Improving health and well-being for infants with prenatal substance exposure. Current Problems in Pediatric and Adolescent Health Care, 54(2), Article 101572. 10.1016/j.cppeds.2024.101572 PMC11025014 38431453

[R11] Channell Doig A, Jasczynski M, Phillips DR, Robinson JL, Aden F, Huq M, Lee K, Jones G, Bernardi C, & Aparicio EM (2023). Experiences of child welfare social workers in addressing substance use among maltreated young mothers to prevent child maltreatment. Child & Family Social Work, 28(3), 846–857. 10.1111/cfs.13009

[R12] Chapman A (2022). The opioid crisis and child maltreatment across counties and time in the United States, 2007–2017. The Annals of the American Academy of Political and Social Science, 703(1), 139–161. 10.1177/00027162221144172 PMC11507506 39464258

[R13] Chuang E, Bunger A, Smith R, (2024). Collaboration strategies affecting implementation of a cross-systems intervention for child welfare and substance use treatment: a mixed methods analysis. Implementation Science Communications, 5, 127. 10.1186/s43058-024-00666-w PMC11552168 39529175

[R14] Chuang E, Ross R, Safaeinili N, Haley LA, O’Masta B, & Pourat N (2025). Collaboration strategies for bridging health, behavioral health, and social services in California’s medical whole person care pilot program. Health Services Research, 60(Suppl 3), Article e14417. 10.1111/1475-6773.14417 PMC12052524 39632564

[R15] Clary E, Ribar C, & Weigensberg E (2020). Challenges in providing substance use disorder treatment to child welfare clients in rural communities.

[R16] Colvin ML, & Howard H (2022). Hard to succeed: A call for social change from mothers with substance use in the child welfare system. Children and Youth Services Review, 140, Article 106574. 10.1016/j.childyouth.2022.106574

[R17] Colvin ML, & Thompson HM (2020). Exploring the experiences of child welfare-focused therapeutic service providers. The Journal of Behavioral Health Services & Research, 47(1), 86–101. 10.1007/s11414-019-09654-8 PMC7224150 30887414

[R18] Cooper RE, Saunders KRK, Greenburgh A, Shah P, Appleton R, Machin K, Jeynes T, Barnett P, Allan SM, Griffiths J, Stuart R, Mitchell L, Chipp B, Jeffreys S, Lloyd-Evans B, Simpson A, & Johnson S (2024). The effectiveness, implementation, and experiences of peer support approaches for mental health: A systematic umbrella review. BMC Medicine, 22(1), 72. 10.1186/s12916-024-03260-y PMC10902990 38418998

[R19] Cooper S, & Nielsen S (2017). Stigma and social support in pharmaceutical opioid treatment populations: A scoping review. International Journal of Mental Health and Addiction, 15(2), 452–469. 10.1007/s11469-016-9719-6

[R20] Dellor ED, Allbright-Campos M, Lee JY, Bunger AC, Gadel F, & Freisthler B (2024). Ohio START: An adaption of the national sobriety treatment and recovery teams model. Children and Youth Services Review, 160, Article 107575. 10.1016/j.childyouth.2024.107575

[R21] Drabble L (2007). Pathways to collaboration: Exploring values and collaborative practice between child welfare and substance abuse treatment fields. Child Maltreatment, 12(1), 31–42. 10.1177/1077559506296721 17218646

[R22] Drabble L (2010). Advancing collaborative practice between substance abuse treatment and child welfare fields: What helps and hinders the process? Administration in Social Work, 35(1), 88–106. 10.1080/03643107.2011.533625

[R23] Drabble L, & Poole N (2011). Collaboration between addiction treatment and child welfare fields: Opportunities in a Canadian context. Journal of Social Work Practice in the Addictions, 11(2), 124–149. 10.1080/1533256X.2011.570657

[R24] Frazer Z, McConnell K, & Jansson LM (2019). Treatment for substance use disorders in pregnant women: Motivators and barriers. Drug and Alcohol Dependence, 205, Article 107652. 10.1016/j.drugalcdep.2019.107652 31704383

[R25] Freisthler B, Michaels N, & Wolf JP (2022). Families in crisis: The relationship between opioid overdoses and child maltreatment in neighborhood areas. Journal of Studies on Alcohol and Drugs, 83(1), 145–154. 10.15288/jsad.2022.83.145 PMC8819892 35040770

[R26] Gopalan G, Kerns SEU, Horen MJ, & Lowe J (2021). Partnering for success: Factors impacting implementation of a cross-systems collaborative model between behavioral health and child welfare. Administration and Policy in Mental Health and Mental Health Services Research, 48(5), 839–856. 10.1007/s10488-021-01135-5 33861385

[R27] Guerrero EG, Marsh JC, Duan L, Oh C, Perron B, & Lee B (2013). Disparities in completion of substance abuse treatment between and within racial and ethnic groups. Health Services Research, 48(4), 1450–1467. 10.1111/1475-6773.12031 PMC3725534 23350871

[R28] Hall MT, Walton MT, Huebner RA, Higgins GE, Kelmel AB, & Lorenz D (2021). Sobriety treatment and recovery teams for families with co-occurring substance use and child maltreatment: A propensity score-matched evaluation. Children and Youth Services Review, 131, Article 106256. 10.1016/j.childyouth.2021.106256

[R29] Hall MT, Wilfong J, Huebner RA, Posze L, & Willauer T (2016). Medication-assisted treatment improves child permanency outcomes for opioid-using families in the child welfare system. Journal of Substance Abuse Treatment, 71, 63–67. 10.1016/j.jsat.2016.09.006 27776680

[R30] Hawk M, Coulter RWS, Egan JE, Fisk S, Reuel Friedman M, Tula M, & Kinsky S (2017). Harm reduction principles for healthcare settings. Harm Reduction Journal, 14(1), 70. 10.1186/s12954-017-0196-4 PMC5655864 29065896

[R31] He AS (2017). Interagency collaboration and receipt of substance abuse treatment services for child welfare-involved caregivers. Journal of Substance Abuse Treatment, 79, 20–28. 10.1016/j.jsat.2017.05.006 28673523

[R32] He AS, Traube DE, & Young NK (2014). Perceptions of parental substance use disorders in cross-system collaboration among child welfare, alcohol and other drugs, and dependency court organizations. Child Abuse & Neglect, 38(5), 939–951. 10.1016/j.chiabu.2013.10.020 24268380

[R33] He AS, & Phillips J (2017). Interagency collaboration: Strengthening substance abuse resources in child welfare. Child Abuse & Neglect, 64, 101–108. 10.1016/j.chiabu.2016.12.011 28064109

[R34] Huebner RA, Hall MT, Smead E, Willauer T, & Posze L (2018). Peer mentoring services, opportunities, and outcomes for child welfare families with substance use disorders. Children and Youth Services Review, 84, 239–246. 10.1016/j.childyouth.2017.12.005

[R35] Huebner RA, Hall MT, Walton MT, Smead E, Willauer T, & Posze L (2021). The sobriety treatment and recovery teams program for families with parental substance use: Comparison of child welfare outcomes through 12 months post-intervention. Child Abuse & Neglect, 120, Article 105260. 10.1016/j.chiabu.2021.105260 34391128

[R36] Huebner RA, Young NK, Hall MT, Posze L, & Willauer T (2017). Serving families with child maltreatment and substance use disorders: A decade of learning. Journal of Family Social Work, 20(4), 288–305. 10.1080/10522158.2017.1348110

[R37] Hwang SHJ, Mollen CJ, Kellom KS, Dougherty SL, & Noonan KG (2017). Information sharing between the child welfare and behavioral health systems: Perspectives from four stakeholder groups. Social Work in Mental Health, 15(5), 500–523. 10.1080/15332985.2016.1252825

[R38] Kelly C, Street C, & Building MES (2022). Child maltreatment 2022. Child maltreatment.

[R39] Lipari RN, & Van Horn SL (2013). Children living with parents who have a substance use disorder. The CBHSQ Report. Substance Abuse and Mental Health Services Administration (US). http://www.ncbi.nlm.nih.gov/books/NBK464590/. 29144715

[R40] Loch SF, Muhar A, Bouskill K, Stein BD, Shi Q, Bonnet K, Schlundt D, Sieger ML, Parker E, Orgel C, & Patrick SW (2023). “The Problem’s Bigger than We Are”: Understanding how local factors influence child welfare responses to substance use in pregnancy, A qualitative study. Child Welfare, 101(2), 193–224. PMC11113001 38784918

[R41] Madden EF, Prevedel S, Light T, & Sulzer SH (2021). Intervention stigma toward medications for opioid use disorder: A systematic review. Substance Use & Misuse, 56 (14), 2181–2201. 10.1080/10826084.2021.1975749 34538213

[R42] Mirick RG, & Steenrod SA (2016). Opioid use disorder, attachment, and parenting: Key concerns for practitioners. Child and Adolescent Social Work Journal, 33(6), 547–557. 10.1007/s10560-016-0449-1

[R43] Ohio University, Voinovich School of Leadership and Public Service. (2023). Parent perceptions and impacts of Ohio START: A qualitative study. https://www.ohio.edu/voinovich-school/research-impact/reports-publications/parent-perceptions-impacts-ohio-start-qualitative-study.

[R44] Ohio START parent graduate follow-up survey: Early findings. (2025). Ohio University Voinovich School of Leadership and Public Service.

[R45] Ohio Attorney General’s Office. (2017, March 22). Attorney General DeWine announces pilot program for families harmed by parental opioid abuse. https://www.ohioattorneygeneral.gov/media/news-releases/march-2017/attorney-general-dewine-announces-pilot-program-fo.

[R46] Olding M, Cook A, Austin T, & Boyd J (2022). “They went down that road, and they get it”: A qualitative study of peer support worker roles within perinatal substance use programs. Journal of Substance Abuse Treatment, 132. 10.1016/j.jsat.2021.108578 34373170

[R47] Orsi R, Boissy L, Yuma P, Palmer F, & Torres-Molinar S (2021). Child welfare in non-metro and rural communities: Experiences of child-serving professionals addressing substance use. Child & Family Social Work, 26(4), 696–707. 10.1111/cfs.12850

[R48] Padilla-Petry P, Fuentes-Pelaez N, & Fantova-Romero A (2025). Navigating ´ relationships and tensions: How professionals who treat substance use disorders view their role in preventing child abuse and neglect. Child Protection and Practice, 5, Article 100164. 10.1016/j.chipro.2025.100164

[R49] Paquette CE, Syvertsen JL, & Pollini RA (2018). Stigma at every turn: Health services experiences among people who inject drugs. International Journal on Drug Policy, 57, 104–110. 10.1016/j.drugpo.2018.04.004 PMC5994194 29715589

[R50] Paterno MT, Fiddian-Green A, & Gubrium A (2018). Moms supporting moms: Digital storytelling with peer mentors in recovery from substance use. Health Promotion Practice, 19(6), 823–832. 10.1177/1524839917750816 29298520

[R51] Patrick SW, Frank RG, McNeer E, & Stein BD (2019). Improving the child welfare System to respond to the needs of substance-exposed infants. Hospital Pediatrics, 9(8), 651–654. 10.1542/hpeds.2019-0106 PMC6663518 31300463

[R52] Radcliffe P (2011). Substance-misusing women: Stigma in the maternity setting. British Journal of Midwifery, 19(8), 497–506. 10.12968/bjom.2011.19.8.497

[R53] Sandfort J (1999). The structural impediments to human service collaboration: Examining welfare reform at the front lines. Social Service Review, 73(3), 314–339. 10.1086/514426

[R54] Schiff DM, Partridge S, Gummadi NH, Gray JR, Stulac S, Costello E, Wachman EM, Jones HE, Greenfield SF, Taveras EM, & Bernstein JA (2022). Caring for families impacted by opioid use: A Qualitative Analysis of Integrated Program designs. Academic Pediatrics, 22(1), 125–136. 10.1016/j.acap.2021.04.016 PMC8542059 33901729

[R55] Seay KD, & McRell AS (2023). Child welfare case managers’ perceptions of parental substance use and experiences with newborn crisis assessments. Child Welfare, 101(3), 25–50.

[R56] Shlonsky A, & Wagner D (2005). The next step: Integrating actuarial risk assessment and clinical judgment into an evidence-based practice framework in CPS case management. Children and Youth Services Review, 27(4), 409–427. 10.1016/j.childyouth.2004.11.007

[R57] Singer SJ, Kerrissey M, Friedberg M, & Phillips R (2020). A comprehensive theory of integration. Medical Care Research and Review, 77(2), 196–207. 10.1177/1077558718767000 29606036

[R58] Stone R (2015). Pregnant women and substance use: Fear, stigma, and barriers to care. Health & Justice, 3(1), 2. 10.1186/s40352-015-0015-5

[R59] Stopka TJ, Estadt AT, Leichtling G, Schleicher JC, Mixson LS, Bresett J, Romo E, Dowd P, Walters SM, Young AM, Zule W, Friedmann PD, Go VF, Baker R, & Fredericksen RJ (2024). Barriers to opioid use disorder treatment among people who use drugs in the rural United States: A qualitative, multi-site study. Social Science & Medicine, 346, Article 116660. 10.1016/j.socscimed.2024.116660, 1982. PMC10997882 38484417

[R60] Tong A, Sainsbury P, & Craig J (2007). Consolidated criteria for reporting qualitative research (COREQ): A 32-item checklist for interviews and focus groups. International Journal for Quality in Health Care, 19(6), 349–357. 10.1093/intqhc/mzm042 17872937

[R61] van Boekel LC, Brouwers EPM, van Weeghel J, & Garretsen HFL (2013). Stigma among health professionals towards patients with substance use disorders and its consequences for healthcare delivery: Systematic review. Drug and Alcohol Dependence, 131(1), 23–35. 10.1016/j.drugalcdep.2013.02.018 23490450

[R62] Wakeman SE, & Barnett ML (2018). Primary care and the opioid-overdose Crisis—Buprenorphine myths and realities. New England Journal of Medicine. 10.1056/NEJMp1802741 29972748

[R63] Wells R, & Chuang E (2012). Does formal integration between child welfare and behavioral health agencies result in improved placement stability for adolescents engaged with both systems? Child Welfare, 91(1), 79–100. PMC3661411 22894016

[R64] Winters AM, Collins-Camargo C, Antle BF, & Verbist AN (2020). Implementation of system-wide change in child welfare and behavioral health: The role of capacity, collaboration, and readiness for change. Children and Youth Services Review, 108, Article 104580. 10.1016/j.childyouth.2019.104580

[R65] Witte TH, Jaiswal J, Mumba MN, & Mugoya GCT (2021). Stigma surrounding the use of medically assisted treatment for opioid use disorder. Substance Use & Misuse, 56(10), 1467–1475. 10.1080/10826084.2021.1936051 34116605

[R66] Yang L, Wong LY, Grivel MM, & Hasin DS (2017). Stigma and substance use disorders: An international phenomenon. Current Opinion in Psychiatry, 30(5), 378–388. 10.1097/YCO.0000000000000351 PMC5854406 28700360

[R67] Yoon S, Ploss A, Hutzel M, Webb R, Hatfield A, Lee JY, Munshi A, Radney A, & McClellan J (2024). Parenting attitudes and behaviors among parents involved with the child welfare system and affected by substance use disorders. Child Abuse & Neglect, 149, Article 106657. 10.1016/j.chiabu.2024.106657 38262180

[R68] Zilberstein K, Brown A, Hatcher AJ, Burton J, & Gau J (2024). Providers’ experiences collaborating with child welfare workers: The good, the bad, and the impacts. Child Abuse & Neglect, 152, Article 106772. 10.1016/j.chiabu.2024.106772 38574602

[R69] Zwick J, Appleseth H, & Arndt S (2020). Stigma: How it affects the substance use disorder patient. Substance Abuse Treatment, Prevention, and Policy, 15(1), 50. 10.1186/s13011-020-00288-0 PMC7385978 32718328

